# Travel-Associated Rabies in Pets and Residual Rabies Risk, Western Europe

**DOI:** 10.3201/eid2207.151733

**Published:** 2016-07

**Authors:** Florence Ribadeau-Dumas, Florence Cliquet, Philippe Gautret, Emmanuelle Robardet, Claude Le Pen, Hervé Bourhy

**Affiliations:** Université Paris Dauphine, Paris, France (F. Ribadeau-Dumas, C. Le Pen);; Institut Pasteur, Paris (F. Ribadeau-Dumas, H. Bourhy);; French Agency for Food, Environmental and Occupational Health and Safety, Malzéville, France (F. Cliquet, E. Robardet);; Assistance Publique Hôpitaux de Marseille, Marseille, France (P. Gautret);; Aix Marseille University, Marseille (P. Gautret)

**Keywords:** rabies, rabies virus, viruses, travel, dogs, cats, humans, risk, communicable disease control, western Europe

## Abstract

In 2015, countries in western Europe were declared free of rabies in nonflying mammals. Surveillance data for 2001–2013 indicate that risk for residual rabies is not 0 because of pet importation from countries with enzootic rabies. However, the risk is so low (7.52 × 10^−10^) that it probably can be considered negligible.

Although western and northern Europe and most countries in central Europe have eliminated rabies in nonflying animals (https://zenodo.org/record/49670#) (*1*,*2*), alerts are regularly issued because of importation of rabid pets. Policy makers recommend postexposure prophylaxis (PEP) after exposure in Western Europe to bats or pet bites in areas with rabies alerts. However, the policy after exposure to these pets is unclear (https://zenodo.org/record/49670#).

Residual risk for rabies in pets in Western Europe is defined as no risk (no PEP necessary) or low risk (PEP recommended after exposure), depending on recommendations (e.g., no risk according to Public Health England and low risk according to the World Health Organization) (*3*). Thus, evaluation of residual rabies risk in western Europe caused by pet movement is needed. We evaluated residual rabies risk caused by pet movement in western Europe.

## The Study

We calculated the risk that a given pet in western Europe is contagious for rabies on a given day by the equation 

We describe factors associated with rabid pets (https://zenodo.org/record/49670#) and define pet transport as any noncommercial movement of a live cat, dog, or ferret and its owner or an authorized person across an administrative border.

During 2001–2013, a total of 21 animal rabies cases attributed to pets from rabies-enzootic countries were reported in western Europe (https://zenodo.org/record/49670#), which represented 1.6 pets/year and 23 days/year of potential contagiousness. Fifteen dogs and 1 kitten originated from rabies-endemic countries outside western Europe. Five dogs raised in western Europe acquired rabies outside this region. One dog subsequently infected 2 indigenous dogs in France (*4*). All pet owners were identified. All owners except 1 (a Spanish man living in a van) were official residents of western Europe. Circumstances that led to pet examination and rabies diagnosis were clinical suspicion (14 pets), bitten humans (3 pets), border quarantine (2 pets), and retrospective data (2 pets with indigenous secondary cases during the alert in France in 2008).

Average contagious period was 16 days/pet: 14 days in western Europe (8 days without signs of rabies and 6 days with signs of rabies) and 2 days before arriving in western Europe. For 1 dog, signs of rabies appeared before the animal entered western Europe. For each rabid animal, an average of 34 (range 0–187) persons and other animals received PEPs. The maximum value of this range corresponds to an alert in France in 2004. After this alert, 1,200 animals were tested and 759 were observed for 1 year. Human and pet vaccinations led to vaccine shortages that required importing of vaccines not authorized for use in France (*5*).

We identified animal origin and mode of entry into western Europe (Table 1). Most rabies cases originated in Morocco and were recorded in France. Three cases were imported from eastern Europe to Germany, 1 from The Gambia to France, and 1 from Sri Lanka to the United Kingdom. Customs officials could not identify any of 11 cases in animals transported mainly by road (e.g., after a ferry trip from Morocco to Spain, Portugal, or France). Seven pets were transported through other countries in western Europe before arriving in the country of diagnosis (https://zenodo.org/record/49670#). Six puppies and 1 kitten were transported by air, of which only 2 were identified by customs officials (in the United Kingdom and Germany).

**Table 1:** Transport mode and origin of 21 pets brought from countries enzootic for rabies to western Europe, 2000–2013

Transport mode	No (%) rabid pets	Country of origin (no.)	Country of diagnosis (no.)
Road	11 (52)	Morocco (9), Croatia (1), Bosnia and Herzegovina (1)	France (8), Germany (2), Spain (1)
Air	7 (33)	Morocco (4), Azerbaijan (1), Sri Lanka (1), The Gambia (1)	Germany (2), Belgium (1), France (2), The Netherlands (1), UK (1)
Unknown	1 (5)	Morocco (1)	Switzerland (1)
None	2 (10)	Dogs from France (secondary cases) (2)	France (2)
**Table 2.** Risk that given dogs or cats are rabid on a given day in 10 countries in western Europe relative to pet transport, 2001–2013*			

Country†	No. pets without signs of rabies				No. days of pet contagiousness for rabies attributed to pet transport								Estimation of probability in the event of contact on a given day with a pet that is contagious for rabies attributed to pet transport		
Pets without rabies signs				Total			Total		
Total	Dogs	Cats		Total	Dogs	Cats	Total	Dogs	Cats	Total, P_A_‡	Dogs, P_AD_§	Cats, P_AC_¶
Belgium	16	16	0		5	5	0		21	21	0		1.38 × 10^–9^	3.32 × 10^–9^	0
France	91	81	10		67	63	4		158	144	14		1.79 × 10^–9^	3.99 × 10^–9^	2.69 × 10^–10^
Germany	32	32	0		31	31	0		63	63	0		9.83 × 10^–10^	2.50 × 10^–9^	0
Ireland	0	0	0		0	0	0		0	0	0		0	0	0
Italy	0	0	0		0	0	0		0	0	0		0	0	0
The Netherlands	1	1	0		4	4	0		5	5	0		2.41 × 10^–10^	7.05 × 10^–10^	0
Portugal	0	0	0		0	0	0		0	0	0		0	0	0
Spain	22	22	0		6	6	0		28	28	0		7.28 × 10^–10^	1.25 × 10^–9^	0
Switzerland	10	10	0		5	5	0		15	15	0		1.62 × 10^–10^	7.10 × 10^–9^	0
United Kingdom	6	6	0		3	3	0		9	9	0		1.18 × 10^–10^	2.37 × 10^–10^	0
Total	178	168	10		121	117	4		299	285	14		NA	NA	NA
Mean	18	17	1		12	12	0		30	29	1		7.52 × 10^–10^	1.57 × 10^–9^#	6.48 × 10^–11^

*NA, not applicable. †We considered only countries in western Europe with a population >1 million persons. ‡P_A_, calculated risk that a given pet is rabid on a given day in a country in western Europe relative to pet transport. §P_AC_, calculated risk that a given cat is rabid on a given day in a country in western Europe relative to pet transport. ¶P_AD_, calculated risk that a given dog is rabid on a given day in a country in western Europe relative to pet transport. #P_AD_ was lower if the dog had no signs of rabies (9.25 x 10^−10^–1.03 x 10^−10^ for dogs with no signs of rabies and 6.44 x 10^−9^–6.44 x 10^−6^ for dogs with signs of rabies assuming that 90%–99.99% had no signs of rabies on a given day).

Of 19 transported rabid pets, 8 (42%) had no rabies vaccination, pet passport, or health certificate. Only 6 were vaccinated (0/2 infected in France, 3/3 imported but raised in western Europe, 3/7 imported by air, and 0/8 imported by road). Most vaccinated pets did not comply with recommended age for vaccination (>12 weeks of age) or time between vaccination, serologic analysis, and transport. No reports mentioned valid rabies serologic analysis included in European Pet Movement Policy (Figure) for unlisted third countries (e.g., Morocco, the Gambia, Sri Lanka, or Azerbaijan) (*6*). Using data for 2001–2013, we calculated that, for contact on a given day with a pet in western Europe, the probability of the pet being contagious for rabies attributed to pet transport was 7.52 × 10^−10^ (Table 2).

**Figure:**
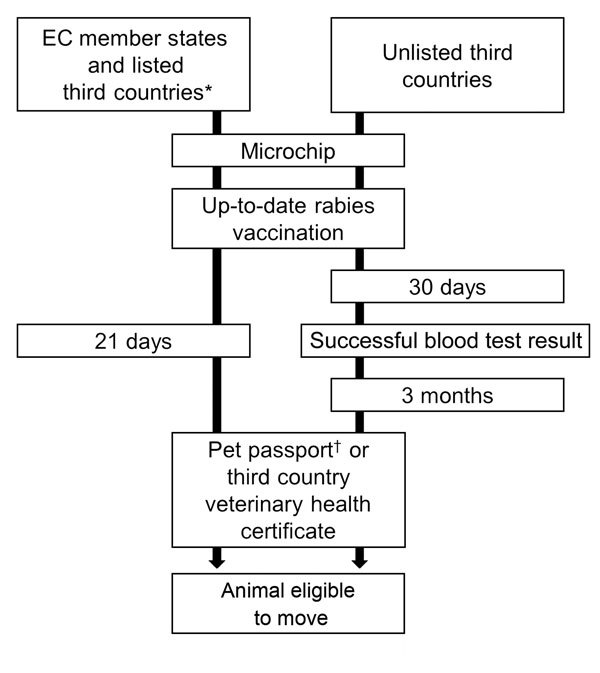
European Union (EU) regulations (no. 998/2003 and no. 576/2013, http://eur-lex.europa.eu/legal-content/EN/TXT/?uri=CELEX:32013R0576) on movement of cats, dogs, and ferrets, 2003–2013. Before 2003, national rules applied (e.g., animal checked at destinations, rabies vaccination, animal identification, quarantine, health certification). EC, European community. *http://ec.europa.eu/food/animal/liveanimals/pets/list_third_en.htm. †A pet passport is required for pets transported in the EU. A health certificate provided by an official veterinarian is mandatory for pets transported from outside the EU.

We observed a significant correlation between number of contagious days for dogs in a country and number of tourists traveling from this country to Morocco (ρ = 0.73, p = 0.017). We found no correlation with other variables tested (total dog population, dog population density, number of dogs per inhabitant).

## Conclusions

Risk for indigenous rabies has decreased in western Europe. During 2001–2013, because of appropriate control of imported rabid pets, only 4 indigenous cases of human rabies were reported (3 in recipients of organs from a donor infected in India and 1 from a rabid bat in Scotland) (https://zenodo.org/record/49670#). Since 2011, no indigenous rabies cases have been reported in terrestrial mammals in western Europe. Because of increased travel (*7*), rabies imported by trips to rabies-enzootic countries has increased, and travel became the main source of rabies in humans (1.46 patients/year) (*8*) and pets (1.6 rabid pets/year) in 2001–2013. However, because of improved surveillance, although the number of imported rabies cases increased, the number of secondary cases decreased (https://zenodo.org/record/49670#).

Illegal importation of rabid animals is not limited to western Europe (*9*) or dogs and cats (*10*). This finding highlights the need for a global approach for regulation of animal movement worldwide and strengthening real-time reporting for animal and human rabies.

Risk for dog rabies being reintroduced into the European Union from Morocco was estimated as 0.21 cases/year (*11*). However, we estimate that 1.1 pets/year are entering western Europe after being infected in Morocco. Morocco has become the main source of pet rabies in western Europe, often through Ceuta and Melilla (Spanish enclaves in northern Morocco). Because no prophylaxis or specific vaccinations are needed for travel to northern Africa, few travelers seek pretravel advice and most have little knowledge of pet rabies (*12*,*13*).

Lack of awareness also increases importation of human rabies. Despite an efficient policy for preventing entry of rabid pets, the United Kingdom reported the highest number of patients with imported rabies during the study period (https://zenodo.org/record/49670#). Patients returning to this country did not believe that a correct PEP was needed after exposure abroad. None of the transported rabid pets fully satisfied European Pet Movement Policy, which raised questions about how to improve the current regulation application. Increasing international travel, expansion of the Schengen area **(**26 countries in Europe that have a common visa policy) into rabies-enzootic countries in eastern Europe, and development of internet animal trade (source of illegal importation) (*14*) are new challenges for ensuring compliance.

Because bat rabies is more difficult to control than dog rabies, and some developing countries still have difficulties controlling rabies, eradication of rabies is not a realistic objective. Awareness should be increased, and current regulations for pet transport should be applied to reduce rabies importation and ensure that risk in western Europe remains low.

To avoid unnecessary and costly PEP and optimize resource allocation, it should be clearly stated which WHO recommendations, Public Health England recommendations, or other practices most relevant after pet exposure should be applied. Low risks (<10-–6) are usually considered acceptable or essentially 0 (*3**,**15*). The risk of a fatal car crash while traveling to PEP consultations was higher than the risk of rabies after exposure to a pet in France in 2001–2011 (*3*). The most pertinent policy in areas at low risk for rabies is probably that of the United Kingdom (i.e., no PEP outside alert areas that do not have asymptomatic animals or exposure to bats) (https://zenodo.org/record/49670#).
